# Consensus Guidelines for the Design and *In Vitro* Preclinical Efficacy Testing N-of-1 Exon Skipping Antisense Oligonucleotides

**DOI:** 10.1089/nat.2022.0060

**Published:** 2023-02-01

**Authors:** Annemieke Aartsma-Rus, Alejandro Garanto, Willeke van Roon-Mom, Erin M. McConnell, Victoria Suslovitch, Winston X. Yan, Jonathan K. Watts, Timothy W. Yu

**Affiliations:** ^1^ Dutch Center for RNA Therapeutics, the Netherlands.; ^2^ Department of Human Genetics, Leiden University Medical Center, Leiden, the Netherlands.; ^3^ N = 1 Collaborative.; ^4^ Department of Pediatrics and Department of Human Genetics, Radboud Institute for Molecular Life Sciences, Radboud University Medical Center, Nijmegen, the Netherlands.; ^5^ Department of Genetics and Genomics, Boston Children's Hospital, Boston, Massachusetts, USA.; ^6^ RNA Therapeutics Institute, UMass Chan Medical School, Worcester, Massachusetts, USA.; ^7^ Harvard Medical School, Division of Genetics and Genomics, Boston, Massachusetts, USA.

**Keywords:** N-of-1, antisense oligonucleotide, exon skipping, protocol

## Abstract

Antisense oligonucleotides (ASOs) can modulate pre-mRNA splicing. This offers therapeutic opportunities for numerous genetic diseases, often in a mutation-specific and sometimes even individual-specific manner. Developing therapeutic ASOs for as few as even a single patient has been shown feasible with the development of Milasen for an individual with Batten disease. Efforts to develop individualized ASOs for patients with different genetic diseases are ongoing globally. The N = 1 Collaborative (N1C) is an umbrella organization dedicated to supporting the nascent field of individualized medicine. N1C recently organized a workshop to discuss and advance standards for the rigorous design and testing of splice-switching ASOs. In this study, we present guidelines resulting from that meeting and the key recommendations: (1) dissemination of standardized experimental designs, (2) use of standardized reference ASOs, and (3) a commitment to data sharing and exchange.

## Introduction

Antisense oligonucleotides (ASOs) offer the unique opportunity for sequence-specific targeting of gene transcripts. This can be exploited to reduce expression of transcripts that give rise to toxic gain-of-function proteins via activation of RNase H, or to modulate RNA splicing to promote or restore expression of partially or fully functional proteins for diseases caused by haploinsufficiency or complete loss of proteins [1]. As such, ASOs offer potential treatment avenues for a plethora of common and rare genetic diseases, in particular those impacting the liver, central nervous system, and retina, tissues to which ASOs can be delivered efficiently. GalNac conjugates allow very robust ASO uptake by hepatocytes after systemic delivery, whereas local delivery is sufficient for broad uptake within the central nervous system and retina (via intrathecal or intravitreal injection, respectively) [2]. Although local treatment is invasive, a low rate of ASO turnover allows for relatively infrequent dosing: every 3–4 months for the central nervous system and 6 months for the eye.

Several oligonucleotides that require intravitreal or intrathecal delivery have received marketing authorization by the Food and Drug Administration (FDA) to treat eye and motor neuron diseases (fomivirsen, Macugen, and nusinersen) [1]. However, it has also demonstrated that it is possible to use ASOs to treat genetic mutations found in as few as an individual patient carrying a unique mutation within an academic setting [3]. Milasen, developed at Boston Children's Hospital, was a mutation-specific ASO for a child with Batten disease who carried an intronic mutation in the *MFSD8* gene that resulted in cryptic splicing.

ASOs targeting the cryptic exon were designed and resulted in restoration of normal splicing and protein production when tested in patient-derived cell lines. After rat safety studies, an investigational new drug application (IND) was filed with the FDA, and investigational treatment with Milasen was initiated less than a year after discovering the pathogenic variant. Milasen administration was associated with a clear drop in the frequency and duration of epileptic seizures and a slower functional decline [3]. However, due to the late stage of her disease, Milasen could not reverse accumulated neuronal damage, and ultimately, the patient passed away 3 years later, although with improved quality of life.

Inspired by this example, initiatives such as n-Lorem were established [4] and also academic groups set out to develop N = 1 ASOs for individual patients such as the Dutch Center for RNA Therapeutics and the “1 Mutation 1 Medicine” (1M1M) initiative [5], and results of academic initiatives to develop individualized ASOs for FUS and C9orf72 amyotrophic lateral sclerosis have been published [6,7]. The N = 1 Collaborative (N1C) was set up as an umbrella organization to align and facilitate individualized treatment development efforts by sharing best practices and learning from successes and failures. N1C aims to provide tools for the different steps involved in N = 1 ASO development such as guidelines for patient/mutation selection, preclinical ASO design and testing, safety and toxicity tests, and regulatory aspects, as well as a toolkit to measure treatment effects in individual patients.

To develop consensus, interactive workshops on specific topics are organized by the N1C. Each workshop is attended by stakeholders involved in N = 1 ASO development: researchers in academia and industry, clinicians, foundations, and patients and their family members. While topics of specific workshops may appeal more to some stakeholders, N1C aims to solicit input from all stakeholder groups to inform and advance best practices for N = 1 ASO development.

This article is the result of an N1C workshop on ASO design and preclinical testing that was held online on July 25, 2022. The workshop covered both exon skipping and RNase H ASOs with the goal of advancing standards for development that are simultaneously rigorous and efficient (reflecting the clinical needs of patients involved in these early efforts, who often have rapidly progressive diseases for which “time is neurons”). This article will focus on the design of exon skipping ASOs, while an accompanying forthcoming article will focus on RNase H ASOs.

## Why Guidelines and Data-Sharing Are Important

While any given mutation-specific ASO may apply to as few as a single patient, this approach could collectively provide benefit to vast numbers of individuals. Successful expansion of this interventional approach will, however, require establishing community standards to ensure rigor in design and development.

Based on the workshop about design and preclinical testing for exon skipping ASOs, we here provide guidelines and considerations. These can benefit preclinical researchers to plan and conduct ASO experiments at a high scientific standard. They also serve as a checkbox to clinicians and patients/families as clinical treatment with an ASO should only be initiated following experiments that were performed properly. This is the first version of these guidelines, based on the current best practices and available knowledge in 2022. We anticipate that with time and increased insight and knowledge, updates will be produced.

Notably, these guidelines focus on proper experimental designs for preclinical ASO development and assume as a prerequisite that due clinical diligence has been performed to assess if exon skipping is expected to be therapeutic in the first place (for more details, we refer the reader to Synofzik *et al.* [8]). Furthermore, the important topic of safety evaluations (to assess if lead compounds are safe before initiating treatment of patients) is beyond the scope of these guidelines. We refer the reader also to the N-of-1+ Oligonucleotide Therapeutics Society briefing document for further information on the full ASO development process: https://www.oligotherapeutics.org/rare-disease-task-force/rare-disease-briefing-document/ and to the N = 1 Collaborative website for up-to-date considerations with regard to safety testing, preparing for clinical trials, and other relevant topics.

## Exon Skipping

**FIG. 1.**
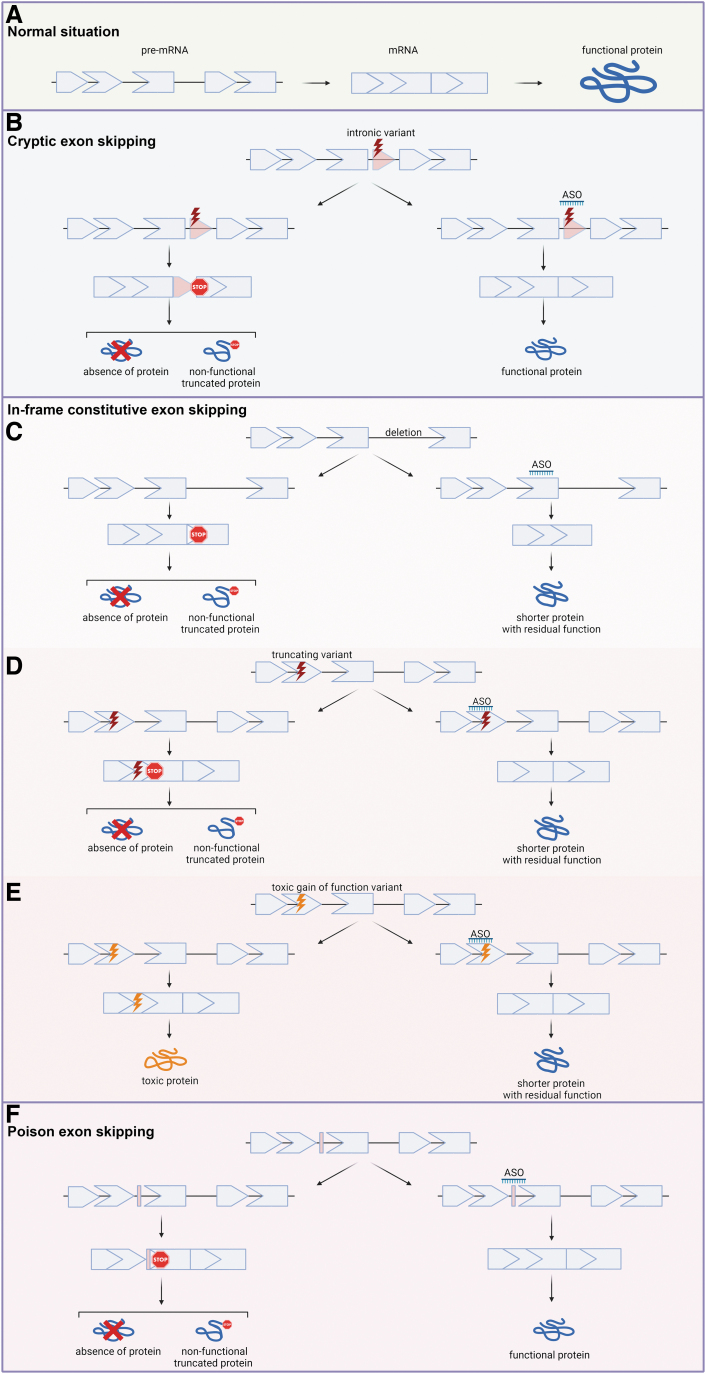
Ways to use ASO-mediated exon skipping to restore protein production. **(A)** Normally, the protein coding information is dispersed over exons in a gene. The gene transcript will contain both exons and introns. During splicing, the introns are removed, resulting in the messenger RNA (mRNA), which is translated into a protein. **(B)** Cryptic splicing variants cause part of an intron to be recognized as an exon and aberrantly included in the mRNA. This prevents protein production. ASOs targeting the cryptic exon can prevent inclusion into the mRNA allowing production of a normal mRNA and normal protein. **(C–E)** ASO-mediated skipping of constitutively spliced exons can restore the production of partially functional proteins in multiple ways: (**C**) by restoring the reading frame, to allow the production of an internally deleted, but partially functional protein; **(D)** by skipping an in-frame exon containing a nonsense or frameshifting variant, which will bypass the variant, while maintaining the reading frame, to allow the production of an internally deleted, but partially functional protein; **(E)** by skipping an in-frame exon containing a toxic gain of function variant, which will allow the production of an internally deleted protein that is partially functional, rather than a toxic protein. **(F)** For some genes, transcripts containing poison exons, short naturally occurring exons containing a stop or a frameshift, are produced. These transcripts are subjected to nonsense-mediated mRNA decay. ASO-mediated skipping of poison exons can increase the amount of functional transcripts produced and thus increase the amount of protein, which can be therapeutic for haploinsufficiency diseases. ASO, antisense oligonucleotide.

There are three general ways in which ASO-mediated exon skipping can be used to treat genetic diseases (Fig. 1). The first is skipping a cryptic or pseudoexon (from here on referred to as cryptic exon) [9]. Variants within the intron have been for long time considered innocuous. However, an increasing number of intronic variants are being described as the cause of rare diseases. These variants can cause part of an intron to be included into the final mRNA, leading to a disruption of the reading frame and therefore reducing protein levels. ASOs targeting these cryptic exons can restore normal splicing and thus normal protein production. Milasen is an example of an ASO targeting a cryptic exon [3]. The advantage of this type of exon skipping is that it restores functional mRNA and protein production.

The second type of exon skipping aims to skip in-frame constitutive exons that harbor a pathogenic variant—most typically, one that causes loss of functional protein, such as a stop gain or frameshift variant [10]. Exon skipping strategies can be designed to bypass the pathogenic variant to allow production of an internally deleted protein. Whether this protein will be (partially) functional will depend on if the skipped exon encodes crucial domains for protein structure or function. For this type of exon skipping, studies to confirm the protein is functional and stable are crucial. One can consider to design ASOs targeting the mutation to achieve exon skipping in an allele-specific manner [11]. However, this will not always be possible, either because the ASOs are not selective enough or because the mutated region is a suboptimal target site for ASO-mediated exon skipping.

Finally, exon skipping can be used for haploinsufficiency diseases by skipping “poison exons” [12]. Poison exons are short, naturally occurring, highly conserved alternative exons that contain a premature termination codon, and when spliced into the transcript targets the transcript for nonsense-mediated decay, leading to reduced protein levels. A current example of using ASOs to increase protein levels by skipping a poison exon is for Dravet syndrome, an autosomal dominant disease, which is caused by variants in the *SCN1A* gene causing reduced expression of voltage-gated sodium channel alpha subunit Na_v_1.1. Interestingly, transcript isoforms containing an exon that disrupts protein production are expressed at relatively high levels in brain. Skipping this “poison exon” increases the amount of functional protein, thus compensating for the haploinsufficiency. This approach is called targeted augmentation of nuclear gene output and restores normal transcripts and proteins. This approach can only be exploited when transcripts with poison exons are produced in the target tissue [13].

Notably, ASO splice modulation can also result in exon inclusion, for example, nusinersen induces the inclusion of exon 7 in *SMN2* transcripts [1]. However, ASOs that induce exon inclusion are harder to develop, typically being dependent on a detailed understanding of the splicing regulatory signals near the exon to be included [14]. In these guidelines, we will only focus on the use of ASOs to induce skipping of cryptic, constitutive, or poison exons.

## Designing Exon Skipping ASOs

Guidelines for the design of exon skipping ASOs have been published based on comparison of effective and ineffective ASOs for dystrophin exon skipping [15]. Furthermore, a software tool to help predict effective exon skipping ASOs has been developed based on published information on exon skipping ASOs [16]. These tools are not yet perfected and are based on limited information. Nevertheless, there are some general rules that can enhance the chance of identifying an effective ASO:

(1) A GC percentage of 40%–60% appears most optimal.
(2) ASOs of 18–22 nucleotides appear most optimal.
(3) Avoid stretches of three or more cytosines in the target sequence, as stretches of three or more Gs in the ASO are very prone to self-structure.
(4) Targeting splicing enhancer sites within an exon increases the chance of efficient exon skipping.
(5) For constitutive exons, targeting the first 30% of the exon and targeting exon–internal sequences rather than splice sites increase the chance of finding an effective exon skipper.
(6) In some cases, effective ASOs do overlap splice sites. We suggest that in these cases the majority of the ASO be designed to target the exon, but with 1–5 nucleotides covering the donor or acceptor splice site. This helps avoid hybridization with unintended targets and helps enable the discovery of ASOs with appropriate GC percentages (although both of those parameters should be checked carefully for splice-site-targeted ASOs).
(7) For pathogenic variants inducing cryptic exons, targeting splicing enhancer sites increases the chances of cryptic exon skipping; however, blocking weak splice sites has also proven effective in some cases

We would like to stress that the guidelines and the eSkip-Finder software will need further optimization and are currently based primarily on skipping constitutive exons. Several studies have confirmed that similar rules apply to cryptic and poison exons [17]. However, the information on these cases is even more limited than for skipping constitutively spliced exons. Larger data sets of effective and ineffective ASOs are required to improve ASO design tools.

Candidate ASOs should be checked for uniqueness in the transcriptome using *in silico* NCBI BLAST analysis. In addition, one should check the genome as well, to rule out overlap with intronic regions and exon–intron junctions. To avoid potential off-target exon skipping, ASOs having homology with other transcripts of 17 or more consecutive nucleotides should be avoided. Assuming the 2′-*O*-methoxy-ethyl phosphorothioate (MOE PS) chemistry of nusinersen is used, ASOs with partial homology of 15 or more consecutive nucleotides to a large number of transcripts should be avoided. While they are unlikely to result in exon skipping, these transcripts may compete with binding to the target transcript resulting in lower efficiency. Note that some third-generation chemical modifications such as locked nucleic acids (LNA) have very high affinity for RNA and likely will already hybridize to shorter homology stretches.

When partial homology with non-target transcripts cannot be avoided, one should consider when and where the target transcript is expressed in relation to the time and tissue that will be treated. For example, if a transcript is only expressed embryonically, it will not be present in patients. Similarly, if the off-target transcript is present only in the central nervous system, while the liver is targeted, this likely will not present a problem.

In addition, the region where the ASOs bind is also important. If an ASO hybridizes directly on an exon or the boundaries, it more likely can affect splicing. In contrast, in the intronic region, chances are lower; yet this should still be verified.

Finally, when one is aware of a potential nonspecific target of an ASO and it is expressed also in the target tissue, one can evaluate in relevant cell types whether the ASO indeed causes exon skipping. Given how challenging it is to develop an effective ASO, it is not likely that ASOs with partial sequence overlap result in efficient exon skipping. However, it can happen and exon skipping events have been reported in cultured cells despite two or three mismatches between the ASO and the skipped exon target sites [18].

If possible, we recommend designing and testing up to 10 ASOs per target exon in initial preclinical studies. This may not be possible if the exon is too short, contains CCC motifs at inconvenient locations, there is homology with other transcripts, or there are repetitive sequences or motifs in the region that are recurrently found in our genes (eg, Alu repeats, microsatellites).

If needed, a second round of ASO design can be performed to optimize ASO efficacy (eg, making the ASOs slightly longer or shorter or moving them 1–2 nucleotides to the left or the right).

## Chemistry Considerations

ASOs with the same chemistry often behave similarly with regard to biodistribution and safety. For this reason, we recommend using MOE PS and 5-methylated cytosine and uridine residues for the development of mutation-specific exon skipping ASOs, that is, the chemical modifications used for nusinersen. For this chemistry, pharmacokinetic and pharmacodynamic data after intrathecal delivery are publicly available for *multiple species* including humans. While the sequence can have profound effects on pharmacokinetic properties and each sequence needs to be tested for safety, the use of MOE PS enables a shorter *in vivo* safety package in a single species (https://www.fda.gov/regulatory-information/search-fda-guidance-documents/ind-submissions-individualized-antisense-oligonucleotide-drug-products-administrative-and-procedural).

However, the 2′-O-methyl phosphorothioate (2OMePS) chemistry without methylated cytosine and uridine residues is a cheaper alternative that generally shows similar efficacy in *in vitro* experiments. As such, it is possible to perform initial target optimization studies with 2OMePS ASOs and to switch to the MOE PS chemistry with methylated cytosines and uridines for lead compounds. In that case, it has to be confirmed that MOE PS ASOs are similarly efficacious *in vitro* before moving to safety studies.

Other chemistries can be considered as well. Whether this is opportune depends on the regulatory jurisdiction. In the United States, N = 1 treatment can only be initiated after an IND filing with the FDA, which involves rigorous safety studies in rodents. However, in Europe, N = 1 treatment can be performed in a “named patient” setting, which does not involve regulatory approval, provided there is a clinical track record for chemical modification used [5]. In that case, not using the nusinersen chemistry (MOE PS) will unduly delay treatment initiation.

## Cell Line Considerations

As the ASOs target human exons, human cell models are needed to establish ASO efficiency, as well as potential off-target effects if identified by BLAST analysis. ASO optimization is ideally carried out in cell cultures that are easy to work with (eg, fibroblasts or neuroblastoma cells rather than neuronally differentiated induced pluripotent stem cells [iPSCs]), but ideally also relevant to the tissue type impacted in disease. When the target exon is not mutated (constitutive or poison exons), wild-type cells can be used. In cases of cryptic splicing mutations or other mutated target exons, patient-derived or gene-edited cells are required.

Obviously, the target transcript has to be expressed in the selected cultured cells. In case of cryptic or poison exons, inclusion of these exons in the transcript has to be confirmed in the cell system used, as these processes can be tissue-specific. In cases where the gene is expressed, but the mutation causes a premature termination codon or an out-of-frame transcript, nonsense-mediated mRNA decay (NMD) can potentially reduce the levels of mutated transcripts rendering it undetectable. In those cases, blocking the NMD process with cycloheximide or a similar reagent will allow the detection of the mutated transcript and therefore allow assessing the efficacy of the ASO in reducing the mutant transcript and increasing the functional one. This is usually the case of cryptic exons, and several studies have shown that the use of cycloheximide revealed the splicing defect, which was not detectable in untreated conditions [19–22].

While iPSCs differentiated to neuronal lineages may not be the first choice due to cost, labor, and time considerations, for some transcripts this will be the only option. Note that transdifferentiation using NGN2 can be an alternative for neuronal differentiation. For other cell types, this might be more difficult. For example, in the retina, although there are protocols for transdifferentiation of fibroblasts to photoreceptor cells using four transcription factors [23], the results are not yet optimal and not all genes of interest are detected. In those cases, alternative models in midigenes for initial screening and patient-derived iPSC cellular models for final validation are required. Notably, minigenes generally are too small to provide sufficient intronic context.

Generally, the use of minigenes or midigenes is not recommended for discovery of exon skipping ASOs because these systems take part of the gene out of its genetic context. Furthermore, the minigenes or midigenes are often expressed in a cell type that is easy to transfect, but where the expression of splicing factors may differ from the cell type where the transcripts are normally expressed. These differences will influence the inclusion of poison and cryptic exons (eg, the cryptic exon is not included in the midigene) [24] as well as ASO efficacy (eg, ASOs are efficacious in the midigene system but not in patient-derived cells of the relevant tissue).

While the use of midigenes and minigenes is not recommended for the reasons outlined above, there will be cases where this is the only option to optimize ASOs in a timely manner. In that case, it is crucial to validate that transcription is similar to the target tissue and to confirm the optimal ASOs in a patient-derived cell setting. Of note, it is important that for cryptic exons caused by deep-intronic variants creating a new splice site, this system has been extremely useful to identify splicing defects in several retinal genes [25]. However, when the insertion of such a cryptic exon was caused by the generation of a splicing enhancer, both midigenes and patient-derived fibroblasts do not always recapitulate the cryptic exon inclusion identified in retinal cells [20,21]. This highlights again the importance of the molecular and genetic context, suggesting that validation in relevant models is required.

## Controls to be Included

### Control considerations

It is crucial to take along control ASOs for various aspects [26]. Controls should have the same chemistry composition as the targeting ASOs. We recommend the following controls in all experiments:

#### Transfection controls

The purpose of these controls is to confirm if the transfection worked properly and may also confirm localization of the ASO (nuclear ideally). Especially if no effective ASO has yet been identified for the target and/or a new cell type is being used, confirming uptake is crucial to avoid concluding that ASOs are ineffective if indeed transfection or delivery failed.

Researchers can use a fluorescently labeled ASO for this purpose, testing localization by microscopy. However, when possible, an ASO known to work efficiently provides the ideal transfection control since it measures functional delivery. This can also target an exon in another transcript and can be the same as the fluorescently labeled ASO mentioned above. If this ASO gives a poor skip, you know that transfection process is not optimal. Even an RNase-H-recruiting ASO targeting a ubiquitously expressed and easy to silence transcript, such as the noncoding RNA MALAT1, can serve as a useful control for efficient transfection.

#### Nontargeting ASO

An ASO that does not target the exon is required to rule out exon skipping due to transfection or ASO treatment *per se* (some alternative splicing may be influenced by this). For functional assays, treatment effects should always be compared with a nontargeting control ASO, as transfection or ASO treatment can influence multiple cellular physiological processes.

It is known that certain ASO motifs can trigger immune responses or change physiological processes in the cells [27]. We therefore recommend using nontargeting ASOs for which it is known that they are well tolerated and do not cause obvious phenotype or gene expression changes in the cells of interest. Nontargeting controls should ideally have a similar GC percentage and length to the targeting ASO, and they should have the same chemical modification.

Different options of nontargeting ASOs exist:

- A previously identified control ASO known to be well tolerated in previous contexts generally does not contain motifs that can trigger immune responses or change physiological processes (may have different GC percentage and length as the targeting ASO)
- Scrambled ASO (will have the same GC percentage and length as the targeting ASO, but may contain undesirable motifs and may target other exons in the transcriptome)
- Sense ASO (ie, complementary to the active antisense sequence) will by definition have the same GC percentage and length as the targeting ASO but may contain undesirable motifs and may target other exons in the transcriptome

We recommend using a previously identified, generic nontargeting control ASO with no known side effects for initial screening studies. However, when functional studies are performed with lead compounds, scrambled and/or sense ASO controls should also be included.

The N1C is currently collecting information to produce an online database of control ASOs with confirmed “good behavior” to facilitate selecting a control ASOs for the cell type of choice.

We recommend using untreated cells as an additional negative control. All the types of nontargeting ASO controls above should show similar readouts relative to the untreated cells: if this is not the case, investigators needs to test additional controls and carry out additional experiments to try to understand what is happening. It may be that the act of transfection or delivery is perturbing the process of interest. The “untreated cells” control is therefore a sort of reality check on the specificity of the effect. However, the nontargeting ASOs are considered the key controls, and when measuring functional effects, the reference sample should be the ASO control rather than untreated cells.

## Transfection Versus Gymnosis

Transfection reagents are very useful to deliver ASOs to cultured cells. We recommend using transfection reagents during the first screening round to assess whether ASOs are effective or not. Generally concentrations of 100 nM or less are sufficient, and efficacy can generally be assessed after 24 h. If you need higher concentrations to induce exon skipping, likely the ASO will not be efficient *in vivo* because the local concentrations in tissues will be lower for most cells. It should be appreciated that transfection of ASOs is extremely efficient and will negate any ASO-specific aspects that can influence efficiency of delivery (eg, length, protein binding).

We therefore recommend to also assess ASO efficacy after gymnotic uptake (naked uptake) for effective ASOs [28]. Here, higher doses of ASOs are needed (0.5–10 μM), and efficacy should be assessed after 72 h or longer. For some cell types, performing gymnotic uptake experiments in 9 mM CaCl_2_ (drastically) improves efficiency, although some cell types do not tolerate this [29].

As gymnotic uptake experiments are generally more predictive of *in vivo* uptake, we recommend basing candidate selection on gymnotic uptake results in relevant models. If gymnosis works very efficiently for the cell model you are using, one could forego the transfection step and only use gymnosis.

## Measuring Efficacy on RNA Level

To measure efficacy and efficiency of exon skipping levels on RNA, RT-PCR analysis can be performed. Several aspects have to be considered here:

### Primer selection

It is possible that, in addition to the target exon, one or more additional exons are also skipped. Therefore, on top of primers targeting flanking exons, primer pairs further away from the target exon should also be included to study this. For larger exons, PCR amplification of the wild-type product might be problematic. The design of primers recognizing either the wild-type exon–exon boundaries versus primers recognizing the novel skipped exon–exon boundaries can be useful in this case. Furthermore, if additional exons are skipped, this can result in out-of-frame transcripts with premature stop codons, which may be quickly degraded and thus go undetected. We suggest, as described above, to use cycloheximide or similar reagents to block NMD and detect these unexpected/undesired transcripts.

### Amplification bias

Smaller fragments will often be amplified more efficiently during PCR than longer fragments. This means that exon skipping levels will generally be overestimated with RT-PCR analysis when based on densitometry analysis of agarose gels. Even a 1% more efficient amplification will result in a 40% increase after 35 cycles (1.4 times overestimation), whereas a 10% more efficient amplification results in a 2,500% increase (25 times overestimation). At the same time, larger fragments will be better labeled and visualized in a gel as they will bind more intercalating dies, creating a bias in the opposite direction. When using Bioanalyzer capillary electrophoresis technology, fragment concentrations will be provided and length will be taken into account [30].

In any case, when different ASOs are compared within the same PCR, the amplification bias will apply to all samples so selecting the most optimal ASOs will still work. However, one should refrain from drawing conclusions on efficiency and efficacy, as this will be a semiquantitative value that can lead to under- or overestimation.

Notably, ASOs will be present in the RNA as well and they will be able to bind to the target exon in transcripts and amplified fragments. This can also interfere with amplification efficiency, especially for ASOs with chemical modifications that result in very high affinity binding to the target (eg, LNA). This will also result in a preferential amplification of the skip fragment as the ASO will only bind to the full-length fragment and not the skip fragments.

### Quantitative polymerase chain reaction or digital droplet PCR

Quantitative polymerase chain reaction (qPCR) analysis can be used to quantify amounts of “non-skipped” and “skipped” fragments. Here, one can normalize for amplification efficiency. Primers have to be carefully designed here to avoid amplification of both the “skipped” and the “non-skipped” fragment for the “non-skipped” primers. One can design primers on the exon–exon junctions to avoid this, but sometimes exon–exon junctions are similar. Therefore, it has to be validated that that the primers are specific only for the intended target (“skipped” or “non-skipped”) with similar efficiencies. As each primer pair can have different efficiencies, this can lead to relative over- or underestimation of the skip, and therefore, efficacy should not be seen as absolute amounts. Also, for qPCR, there can be interference of ASOs binding to transcripts and amplification fragments containing the targeted exon.

The golden standard using absolute quantification is the digital droplet PCR (ddPCR) [30] or RNA-sequencing. However, these are expensive techniques and may not be available to everyone. There are cases, however, where ddPCR or RNA-sequencing may be the only option (eg, to discriminate between two transcripts of similar length). For ddPCR, one will also have to confirm specificity of the ddPCR probes for skip and non-skip products. Furthermore, while this provides an absolute quantification, one should realize that *in vitro* studies do not directly translate to the *in vivo* situation, where the efficacy likely will be lower.

Pragmatically, we recommend using standard RT-PCR to compare ASO efficiencies and select lead candidates if possible. We do urge users to be careful when mentioning exon skipping percentages as a quantitative measure, and to clearly state this is only semiquantitative and likely an overestimation of the real situation.

When cycloheximide treatment is needed to observe non-productive transcripts, each condition should be performed with cycloheximide treatment. Only the nontreated condition needs to be performed both with and without cycloheximide. This will also serve as validation of those transcripts appearing upon inhibiting NMD. For cryptic exon containing transcripts subjected to NMD, blocking NMD will allow assessment of a decrease of the aberrant transcript in parallel with assessing an increase of the wild-type transcript.

## Assessing Effects on Protein and/or Functional Level

The studies to assess protein restoration or reduction of functional deficits will depend on the protein involved and the cellular function of this protein. As such, we cannot make detailed recommendations. However, a common procedure we suggest to perform for lead candidates is first confirming protein restoration. In cases where protein restoration is detected, additional studies to assess protein stability and normalization of functional deficits can be performed as well. Protein analysis relies on the availability of antibodies and the most straightforward test is Western blot analysis. For approaches aiming to skip an in-frame exon, Western blot analysis can also confirm the production of a shorter protein. We realize that, in some cases, performing these studies will not be possible (eg, because no antibody is available, the protein is difficult to blot, or because no deficits are seen in cultured cells).

When these experiments are possible, inclusion of the proper controls (see above) is crucial to avoid drawing false-positive or false-negative conclusions. In these cases, the functional effects have to be offset to the control ASO reference. However, as mentioned previously, taking along an untreated sample as well is recommended to assess whether the treatment procedure has an effect on protein or function *per se*. Many of the confounding effects described above (such as PCR artifacts and primer or ASO interference) do not apply to a study of protein-level expression, so positive protein-level data are of significant help in building confidence that the ASO is performing as expected.

## Takeaway Messages for Clinicians and Families

These guidelines aim to facilitate exon skipping ASO development in a standardized and scientifically rigorous manner to allow obtaining high-quality efficiency and efficacy data, which will be useful to optimize software for exon skipping ASO design. However, there are also takeaway messages for clinicians who will be provided with an ASO to treat a patient and patients and families who are given the possibility to be treated by such an ASO.

It is clear that developing and optimizing ASOs preclinically is complicated. However, toward N = 1 treatment, a collaborative effort is required, involving different perspectives that are not all covered by preclinical researchers' expertise. Clinicians need to ask critical questions about using exon skipping as a therapeutic option for a specific variant, patient, and disease: *Does the rationale make sense? Will exon skipping indeed lead to a functional protein? Will this result in clinical benefit for the specific patient? What will this benefit look like and it is possible to measure it? Is it possible to treat this patient with intrathecal injection or does the pathology prevent this (eg, scoliosis)? What would that benefit look like?* Patients and families should also question whether the expected benefit would outweigh the burden of a repetitive invasive treatment.

While it may not be possible for clinicians and patients and families to understand the nuances of the preclinical studies, checking if quality controls were included should be possible. Also the level of detail can be easily assessed: *does the preclinical work involve only RNA analysis or demonstrates protein-level rescue and functional effects of treatment?* Demonstrating functional effects, when possible, is obviously of higher value in terms of showing promise for translation to further development toward patient use. When a researcher provides an ASO for clinical use in patients, both patients and clinicians should question if relevant safety studies were conducted.

## Recommendations for the Future

There are three takeaways from our meeting. First, it is crucial to disseminate best practices, which we aspire to do with this publication. Second, it is important to have standardized reference ASOs, which is a resource the N1C will compile. Finally, data sharing will be crucial: N1C will only be able to achieve its goals if we implement data sharing standards to allow each effort to inform the next, supporting a continual improvement process that maximizes efficiency, efficacy, and safety. Sharing these data will allow critically important retrospective analyses of ASO efficacy and safety and enable the derivation of open-source computational design algorithms to further support the community. The quality of the design software will depend on the quality of the data submitted. Ideally, studies are performed with scientific rigor, and all required positive and negative controls were included, which brings us back to the importance of best practices.

Developing N = 1 ASOs takes the combined and active participation of a group of experts, including preclinical researchers, clinicians, genetic counselors, patients, pharmacological and toxicology experts, hospital pharmacists, regulators, and ethicists. While a team of people is needed for each specific N = 1 ASO and each individual patient, processes and procedures can be streamlined across efforts. The N1C aims to provide best practices and guidelines to give each of the stakeholders the tools to be actively involved and to facilitate development of N = 1 ASOs for the patients who need them. We also welcome you to submit your effective and ineffective ASO sequences to the N = 1 database once it comes online. The preclinical design and development of exon skipping ASOs is a small part of this process, but we hope that the guidelines will be useful to those developing N = 1 ASOs.
